# Complaints against health professionals regarding patients’ suicidal thoughts and behaviours: retrospective study of disciplinary cases in The Netherlands

**DOI:** 10.1192/bji.2025.10051

**Published:** 2025-11

**Authors:** Wilco C. Janssen, Tom C. Snijders, Frank L. Gerritse, Sisco M. P. van Veen

**Affiliations:** 1 113 Suicide Prevention, Amsterdam, The Netherlands; 2 Department of Psychiatry, Tergooi Medical Centre, Hilversum, The Netherlands; 3 Department of Psychiatry, Amsterdam University Medical Centre, Amsterdam, The Netherlands. Email: s.vanveen4@amsterdamumc.nl

**Keywords:** Disciplinary law, suicidality, complaints, litigation, health professionals

## Abstract

**Background:**

Suicidal thoughts and behaviours (STBs) are common within healthcare systems. Diagnosing and treating them is challenging for healthcare professionals. Therefore, the way they respond to patients’ STBs constitutes regular grounds for complaints filed against them. Studies on disciplinary complaints regarding STBs are scarce and thus far have exclusively focused on death by suicide and primarily investigated psychiatrists.

**Aims:**

To gain more insight into disciplinary law cases concerning patients’ STBs in The Netherlands.

**Method:**

A total of 108 public cases between 2010 and 2021 were codified and analysed.

**Results:**

Most complaints concerned undertreatment and insufficient involvement of the patient’s relatives or other healthcare professionals. Nearly half of the complaints were filed against psychiatrists.

**Conclusions:**

Overall, compared with the number of health professionals in The Netherlands, risk of litigation appeared to be very low. Further research could be conducted on the discrepancy between the number of founded and unfounded complaints in first-instance and appeal cases.

Worldwide, more than 720 000 people die by suicide each year, often alone and in despair.^1^ On average, each suicide affects the lives of 135 people, on whom it can have a profound impact.^2^ Witnesses and relatives often experience feelings of sadness, guilt, anger and rejection and are at increased risk for a range of mental health problems, such as complicated grief and post-traumatic stress disorder.^3^

Many people with suicidal thoughts and behaviours (STBs) come into contact with the healthcare system. According to a systematic review, 31% of people who died by suicide had been in contact with a mental health professional in the year prior to their death and 80% had visited their general practitioner.^4^ Understandably, there is a strong appeal from patients, relatives and society at large that health professionals prevent suicide. However, health professionals are limited in their ability to do so. For example, although it is common practice (and often required) for health professionals to conduct a risk assessment, research has consistently shown that it is impossible to predict which person will attempt suicide and when: the most optimistic estimates suggest that suicide risk assessment will miss approximately 44% of all cases, while resulting in 94.5% false positives.^7^ Furthermore, there are very few evidence-based interventions for STBs and although the interventions available have almost exclusively been found to reduce suicidal thoughts or suicide attempts, their ability to prevent suicide remains unknown.^8,9^

Because of this discrepancy between the expectations and possibilities of professionals, it is understandable that the way health professionals respond to patients’ STBs regularly results in complaints filed against them. For instance, Frierson & Joshi reported on 30 years of malpractice claims, administered by a prominent malpractice insurer in the USA, and found that 27% of all such claims brought against psychiatrists involved suicide or a suicide attempt.^10^ In The Netherlands, Gerritse & Duvivier analysed all medical disciplinary cases against psychiatrists and residents in psychiatry between 2015 and 2019 and found that 5.7% of the complaints involved suicide.^11^

The disciplinary law system varies for most countries, reflecting differences in legal traditions, governance structures and societal expectations of accountability. For instance, the Dutch system emphasises quality assurance and public trust through measures such as warnings or suspensions, while excluding financial compensation or criminal penalties, which contrasts sharply with systems like those in the USA or Canada, which are more litigation-driven and often include financial settlements.^12^ Additionally, the Anglo-American focus on adversarial proceedings differs from the more consensus-oriented and rehabilitative approaches seen in European systems, highlighting the need for careful contextual interpretation of disciplinary cases.

In this article we explore the Dutch context, where disciplinary law differs from civil law in that complainants cannot file for financial compensation and defendants cannot be given prison sentences or other penal punishments by the disciplinary tribunals. The assumption is that any ‘directly interested party or stakeholder’ can file a complaint against any registered healthcare professional. This stakeholder is often a patient, their partner or a close relative. Likewise, an employer or healthcare institution can also file a complaint. All complaints are handled by one of the five regional disciplinary tribunals. After a regional tribunal has made a decision, both the complainant and the defendant can appeal to a central disciplinary tribunal for healthcare. If the complaint is substantiated and the healthcare professional is found to be at fault, a number of disciplinary measures can be demanded, including: a formal warning or reprimand, a fine (paid to the state), probation, or a suspension (temporary or permanent) from practice. For a more elaborate description of Dutch disciplinary law, see Gerritse & Duvivier.^11^

Studies of complaints against mental healthcare professionals regarding patients’ STBs have thus far focused almost exclusively on death by suicide, not on suicide attempts or suicidal thoughts; they are often over 30 years old and have mainly investigated complaints against psychiatrists, not other mental health professionals. Furthermore, almost all studies to date have been conducted in the USA.^10,13,14^

An update and expansion of this literature base is needed. Learning from recent disciplinary cases in different jurisdictions can help identify universal areas for improvement in the care for people with STBs. Knowing what types of clinical situation typically lead to complaints may help to identify areas of improvement that are relevant in all jurisdictions. For instance, it may reduce the clinical decision-making based on fear of litigation that can lead to overly restrictive and coercive measures or avoidance of people with STBs.^15^ Therefore, this study aims to analyse the number, nature and outcomes of disciplinary complaints against healthcare professionals in The Netherlands.

## Method

### Data collection

#### Ethical approval and privacy

Since we used publicly available and anonymised data, no ethical approval or informed consent was needed for this study.

#### Information sources

Anonymised versions of all disciplinary cases in The Netherlands are in the public domain and accessible through https://tuchtrecht.overheid.nl. A Python script using the Scrapy framework was developed to systematically collect cases from the website. The script searched cases using keywords corresponding to healthcare professions (e.g. the Dutch words for ‘physician’, ‘nurse’, ‘psychotherapist’) within a specified time frame (2002–2021). For each case, relevant details, such as case number, profession, date of judgment and decision, were extracted and stored in a structured format (JSON). Only publicly available, anonymised cases were included, ensuring compliance with privacy and ethical standards. The source code is available online at https://github.com/flgerritse/STB_disciplinary_law. Rulings contain a summary of the complaint, the complainant’s and defendant’s point of view, the considerations of the tribunal and its verdict.

Healthcare professionals as defined in the Healthcare Professionals Act^16^ were compared with the total number of healthcare professionals registered in their category and the total number of suicides as registered by the Dutch Central Bureau for Statistics.^17^ As the number of registered professionals changes each year, the most recent data available at the time (from 2020) were used.^18^

#### Search strategy and eligibility criteria

**Fig. 1:**
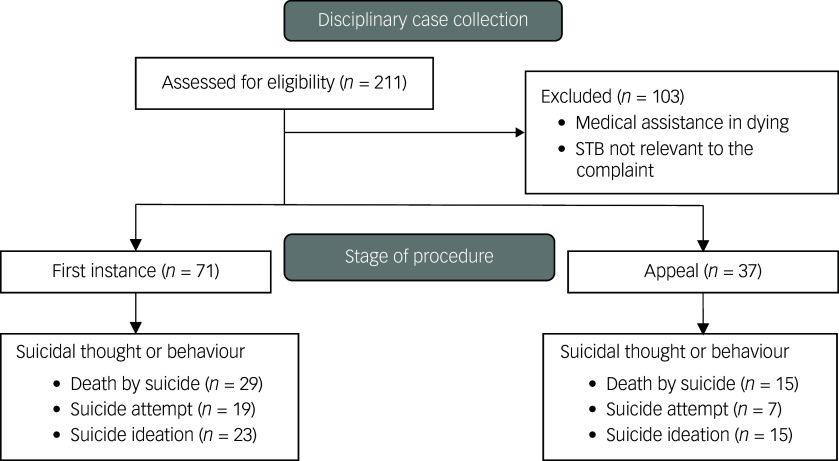
Flow diagram. STB, suicidal thought and behaviour.

We selected all cases in which a reference was made to STBs by searching for the keyword ‘suicid*’, and the Dutch synonyms for suicide ‘*zelfdoding*’ and ‘*zelfmoord*’. We collected all disciplinary law cases in The Netherlands published between 1 January 2010 and 31 December 2021 in which the complaint concerned the response of the defendant to a patient’s STBs (Fig. 1). The inclusion criterion was: all reports regarding STBs. The exclusion criterion was: all cases published in 2021 that were not appeals. This to ensure that both the first instance and a possible appeal were included in the data.

#### Selection process

Cases were screened for eligibility criteria using ATLAS.ti 22 for MacOS (ATLAS.ti Scientific Software Development GmbH, Berlin, Germany; https://atlasti.com) by one of the authors (T.C.S.), under the supervision of a licensed healthcare psychologist and researcher (W.C.J.). First-instance and appeal cases were separated based where they were served: cases handled by the regional disciplinary board were considered cases at first instance (first-instance cases) and cases handled by the central disciplinary board were considered appeal cases.

#### Coding

Prior to coding, a list of possible codes was constructed. New codes were added during the coding process at the discretion of the coder (T.C.S.). After coding had been completed, the coder and supervisor (W.C.J.) reviewed the codebook and combined codes that were not clearly distinguished from each other or provided little extra information (i.e. iterative sampling; see Supplementary Tables S1, S2 and S3, available online at https://doi.org/10.1192/bji.2025.10051).

## Results

### Complaints and type of STBs

Between 2010 and 2021, a total of 255 complaints (Table 1) regarding STBs were filed against mental health professionals across 71 cases at first instance. An additional 118 complaints were filed across 37 cases in appeal. At first instance, 29% of the complaints were founded. Roughly 16.1% of complaints were upheld at appeal.

**Table 1:** Complaints at first instance and appeal

Complaint	Total, *n* ^a^	First instance		Appeal	
		Founded, *n* (%)	Unfounded, *n*	Upheld, *n* (%)	Overturned, *n*
Undertreatment	44	13 (29.5%)	31	3 (21.4%)	11
Too little collaboration with patient’s system	31	9 (29%)	22	2 (18.2%)	9
Too little collaboration with another health practitioner	26	6 (23.1%)	20	1 (9.1%)	10
Misjudged suicide risk	24	10 (41.6%)	14	3 (27.3%)	8
Underdiagnosis of psychiatric disorders	22	7 (31.8%)	15	0 (0%)	13
Inadequate documentation	21	5 (23.8%)	16	0 (0%)	15
Incorrect/risky prescription of medicine	20	4 (20%)	16	0 (0%)	6
Inadequate risk assessment of returning patient to country of origin – potential risk of STBs	17	4 (23.5%)	13	1 (11.1%)	8
Insufficient/inappropriate communication with the patient or their system	11	2 (18.2%)	9	0 (0%)	2
Insufficient care for surviving relatives	10	4 (40%)	6	1 (33.3%)	2
Overtreatment	8	1 (12.5%)	7	0 (0%)	2
Insufficient knowledge of patients’ medical records	7	2 (28.6%)	5	0 (0%)	0
Inadequate execution of STB treatment	6	1 (16.7%)	5	1 (33.3%)	2
Shared too much information with another health practitioner	6	1 (16.7%)	5	1 (25%)	3
Did not provide sufficient supervision/intervision	5	3 (60%)	2	1 (50%)	1
Did not seek sufficient supervision/intervision	4	3 (75%)	1	0 (0%)	1
Unacceptable behaviour – personally involved with the patient	4	3 (75%)	1	3 (100%)	0
Failed to inquire informed consent	3	1 (33.3%)	2	0 (0%)	3
Used information from an unreliable source/person	3	0 (0%)	3	0 (0%)	0
Decision-making beyond area of expertise	2	1 (50%)	1	0 (0%)	0
Incorrect referral	2	0 (0%)	1	0 (0%)	1
Inexperienced practitioner	2	0 (0%)	1	0 (0%)	1
Insufficient treatment of something other than STBs	1	1 (100%)	0	1 (100%)	0
Unpleasant physical examination	1	0 (0%)	1	0 (0%)	1

STBs, suicidal thoughts and behaviours.

a. Only first-instance complaints are shown in the ‘Total’ column.

Most complaints at first instance followed death by suicide (41%) (Fig. 1) and concerned undertreatment (16%) or insufficient collaboration with either relatives (11%) or other healthcare professionals (9%) (Table 1). These were followed by complaints about the assessment of STBs (9%) or underdiagnosis of psychiatric disorders (e.g. failure to recognise that the patient was suffering from psychosis; 8%), about inadequate documentation (8%) and about the prescription of medication (7%). A few complaints concerned inappropriate communication (4%), insufficient care for surviving relatives (4%) and overtreatment (3%).

Most complaints overall (first instance and appeal) were in reaction to a suicide (41%), followed by complaints concerning suicide attempts (35%) and suicidal ideation (24%; Fig. 1).

### Complainants

Most complaints were filed by patients (*n* = 62; 57%), followed by surviving relatives (*n* = 40; 37%). Complaints from relatives (*n* = 5; 5%) and the healthcare inspectorate (*n* = 1; 1%) regarding professionals’ dealings with STBs were less common.

Complainants responsible for two or more disciplinary cases were considered ‘serial litigants’. Serial litigants (*n* = 10) represented 11% of the total complainants, but were responsible for 29% (*n* = 74) of the total complaints and 25% (*n* = 27) of the total disciplinary cases. On average, 24% (*n* = 43) of the total complaints were founded. For the serial litigants, this number was lower (*n* = 7; 10%).

### Defendants and work setting

Nearly half of all complaints were filed against psychiatrists (49%) (Table 2). There was also a substantial number of complaints against medical advisors (19%) of the Immigration and Naturalisation Service (INS). Usually these are non-specialty doctors. These complaints were predominantly filed by people with suicidal ideation who complained that the medical advisor underestimated their suicide risk if they were to be deported to their country of origin, for instance because of the stress this would impose on them or the unavailability of mental health treatment there. There were 14 complaints filed against general practitioners. Complaints against nurses (7%) and psychologists (6%) were rare.

**Table 2:** Complaints by profession

Profession	*n*	Total complaints, *n* ^a^	First instance		Appeal	
			Founded, *n* (%)	Unfounded, *n*	Founded, *n* (%)	Unfounded, *n*
Psychiatrist	53	122	44 (36.1%)	78	4 (8.9%)	41
Medical advisor	21	45	11 (24.4%)	34	3 (10.3%)	26
General practitioner	14	38	5 (13.2%)	33	1 (10%)	9
Nurse	8	24	6 (25%)	18	2 (22.2%)	7
Psychologist	7	17	8 (47.1%)	9	9 (47.4%)	10
Insurance physician	3	6	0 (0%)	6	0 (0%)	4
Company doctor	2	3	0 (0%)	3	0 (0%)	2

a. Only first-instance complaints are shown in the ‘total’ column.

In 2020, out of the total 3845 registered psychiatrists, 5 (0.13%) received a complaint regarding STBs. Only 1 (0.004%) out of the total 21 950 registered psychologists received such a complaint and only 1 (0.0004%) of the total 204 410 nurses. Furthermore, in 2020, there were a total of 1825 suicides reported in The Netherlands. Only 7 of these deaths (0.38%) resulted in complaints.

Most defendants worked for in in-patient (*n* = 29) or out-patient clinic (*n* = 24). A relatively small number of defendants worked in a crisis team (*n* = 8), general hospital (*n* = 6) or insurance agency (*n* = 3).

### Appeals

A total of 37 appeal cases were recorded. Out of the 37 appeals, 21 were made by the complainant and 16 by the defendant.

Only one complainant had a successful appeal. This appeal resulted in a formal warning for the defendant. For the 20 other complainants, their appeal was unsuccessful.

A total of 11 defendants had a successful appeal, leading to a repeal of the disciplinary measure imposed on them. Six of the successful appeals were related to psychiatrists who appeared to have acted in accordance with care they should have given to the patient. Four of the appeals involved INS medical advisors, who were not expected to be capable of a risk analysis for their client. Additionally, one INS medical advisor was accused of passing on contradicting advice. During appeal it was argued that the two accounts of the advice were 2 years apart other and well-supported.

Four defendants were not successful in their appeal and their formal warnings remained unchanged. One defendant, a psychologist, appealed the decision in an attempt to change the disciplinary measure of a 1 year suspension to a probationary period. She was accused of failing to provide adequate treatment and personally involving herself with the patient. The tribunal deemed her defence to be insufficient and was not convinced by her argument that she had learned from the case. Instead, they considered the psychologist unable to practise her profession again and permanently revoked her registration in the Healthcare Professionals Act register.

## Discussion

The objective of this study was to provide insight into the number, nature and outcomes of disciplinary cases concerning patients’ STBs, filed against health professionals from a wide range of professions in The Netherlands.

### Risk of litigation

Our analysis shows that the risk of disciplinary complaints against healthcare professionals in The Netherlands concerning suicidality is very low. Hardly any complaints were filed against certain professional groups. For example, over a period of 11 years, only 7 psychologists and 8 nurses received a complaint. Complaints against psychiatrists were more frequent, probably because they often serve as lead clinicians in settings where STBs are more common. Nevertheless, these were still rare: in 2020, only 53 psychiatrists (0.13%) received a complaint related to suicidality.

Given the rarity of such complaints and the small size of this professional group, it is noteworthy that approximately 15% of the professionals receiving a complaint worked as medical advisors for the INS. In each of these cases, complainants facing deportation argued that the medical advisor had overestimated the availability of adequate treatment options in their country of origin, potentially increasing suicide risk.

### Areas for improvement

The most common reason for a disciplinary complaint concerned inadequate collaboration between healthcare providers and relatives or other healthcare professionals. Approximately 1 in 5 complaints were about this subject. This suggests that improvements in this area are possible. For example, apart from stating that it is important to engage relatives, guidelines and training programmes would do well to offer concrete examples of how this might be done and include clear information about how it relates to professional confidentiality. Previous studies^10^ in other countries have not identified this type of complaint as particularly prevalent, likely because they examined malpractice claims, which focus on establishing a causal link between the care provided and the patient’s death. In contrast, the Dutch disciplinary law system adopts a broader perspective, assessing overall professional conduct.

Other frequent complaints concerned the professional misjudging suicide risk or not having taken sufficient action to mitigate this risk. This is noteworthy, as extensive research has shown that the predictive value of such risk assessments is highly limited: according to meta-analysis, risk assessments by clinicians fail to identify 44% of the people who die by suicide in the years after the assessment and they result in 94.5% false positives.^7^ Professionals are not able to predict to a meaningful extent who will die by suicide.

This is not to say that professionals have no role in suicide prevention. Several brief interventions have been shown to reduce the risk of suicidal behaviour, such as safety planning,^19^ proactive follow-up contacts^20^ and adequate treatment of mental health problems.^21^ It seems reasonable to expect professionals to provide these and other effective interventions to everyone in high-risk groups, such as individuals recently discharged from psychiatric hospital, as well as to everyone with STBs, since these are considered precursors to suicide and therefore warrant precaution.^22^ Offering such interventions to everyone in high-risk groups, regardless of their presumed suicide risk, could lead to a substantial reduction in suicidal behaviour without requiring clinicians to predict who will attempt suicide or not.

### Functioning of the Dutch disciplinary law system

With regard to the functioning of the Dutch disciplinary law system, it is noteworthy that there is a notable discrepancy between the number of founded complaints at first instance and appeal. Additionally, the number of complaints coming from ‘serial litigants’ was remarkable: these complainants were responsible for a third of the complaints and a quarter of all cases. To better protect professionals against unfounded or excessive accusations, it may be worth considering reforms that raise the threshold for disciplinary proceedings. A stepped approach that promotes pre-complaint resolution could be helpful, involving mandatory triage to determine whether a complaint is appropriate for the disciplinary tribunal or better suited for a lower-level complaints body.^23^ Additionally, limiting the statutory grounds for disciplinary action might help ensure that only complaints about sufficiently serious or persistent errors proceed to a disciplinary tribunal. At present, Dutch disciplinary law allows complaints for any conduct deemed contrary to the care that befits a good caregiver, which is a broad and sometimes vague standard. This may lead to full disciplinary proceedings over relatively minor or isolated issues, rather than focusing on more serious or persistent shortcomings in care. A clearer specification of the standard could help ensure that only substantively concerning cases are escalated to the disciplinary tribunal.^24^

### Limitations

The Dutch disciplinary system is inherently national, which makes it difficult to apply these findings to other countries. Furthermore, some complaints may have been handled within the disciplinary systems of professional associations, such as the psychologists’ association, and therefore were not published on the government website from which our data were drawn. Owing to the small numbers, it was not feasible to report detailed findings separately for different types of STB.

### Future directions

Larger studies combining data from multiple jurisdictions are warranted. Given that a substantial proportion of the complaints concerned collaboration with relatives, further research into the facilitators and barriers professionals encounter when engaging with relatives is also important.

## Supporting information

Janssen et al. supplementary material Janssen et al. supplementary material

## Data Availability

The case files used in this study are openly available at https://tuchtrecht.overheid.nl/ (in Dutch). The codebooks can be found in the Supplementary Tables. The selected and codified case files are available from the corresponding author (S.M.P.v.V.) on reasonable request.

## Supplementary material

The supplementary material is available online at https://doi.org/10.1192/bji.2025.10051.
