# Supplementary material for: Complaints against health professionals regarding patients’ suicidal thoughts and behaviours: retrospective study of disciplinary cases in The Netherlands
Source: BJPsych Int. 2025 Aug 15;22(4):113–9. doi: 10.1192/bji.2025.10051 (PMC13054158; doi:10.1192/bji.2025.10051)
Supplement: Janssen et al. supplementary material [file S2056474025100512sup001.docx]

| Table A1. Clustered codes |  |  |
| --- | --- | --- |
| Code |  | Example of original code |
|  |  |  |
| Undertreatment |  | Limited and inadequate treatment of the patient |
|  |  | Late referral |
|  |  | Insufficient monitoring of the patient |
|  |  |  |
| Misjudged suicide risk |  | Incorrect carried out risk assessment |
|  |  | Insufficient monitoring of STBs |
|  |  | Insufficient diagnostics of STBs |
|  |  |  |
| Too little cooperation with patient’s system |  | Shared too little information with patient’s relatives |
|  |  | Too little information used from patient’s relatives |
|  |  | Failed to share by the complainant (relative) requested records |
|  |  |  |
| Too little cooperation with another health practitioner |  | Too little information used from another health practitioner |
|  |  | Insufficient transfer of patient |
|  |  | Shared too little information with another health practitioner |
|  |  |  |
| Insufficient diagnostics about something other than STB |  | Failed to perform assessment of something other than STBs |
|  |  | Failed to perform additional (requested) diagnostics |
|  |  |  |
| Overtreatment |  | Overtreatment with malicious intent |
|  |  | Overtreatment – exacerbating STBs |
|  |  |  |
| Failed to inquire informed consent |  | Diagnostics without consent by a parent/guardian |
|  |  | Failed to ask the patient for consent |
|  |  |  |
| Insufficient/inappropriate communication with the patient or system |  | Insufficient communication with the patient |
|  |  | Inappropriate communication with patient’s relatives after death of the patient |
|  |  |  |
| Inadequate documentation |  | Incorrect reports provided |
|  |  | Incorrect use of medical terms |
|  |  | Lying about / making up of medical record |
|  |  |  |

**Appendices**

| Table A2. Codebook for complaints |  |  |
| --- | --- | --- |
| Code |  | Example from disciplinary case |
|  |  |  |
| Decision making beyond area of expertise |  | Did not limit his/her advice to their own expertise |
|  |  | Should not have opted to assess incapacitation |
|  |  |  |
| Did not provide sufficient supervision/intervision |  | Insufficient guidance of nursing staff |
|  |  | No agreement with subordinates to monitor patient |
|  |  |  |
| Did not seek sufficient supervision/intervision |  | Did not seek an expert opinion |
|  |  | Sedated the patient without consulting the leading psychiatrist |
|  |  |  |
| Failed to inquire about informed consent |  | Did not inform the patient on the proposed examination or request permission |
|  |  |  |
| Inadequate documentation |  | Medical record does not meet professional standards |
|  |  | Added incorrect information to the medical record |
|  |  |  |
| Inadequate execution STB treatment |  | Inadequate carried out cognitive therapy for STB |
|  |  | Inadequate psychiatric aid regarding STB |
|  |  |  |
| Inadequate risk assessment of returning patient to country of origin – potential risk of STB |  | Did not take into account the circumstances in country of origin |
|  |  | Incorrect assessment of treatment continuity in country of origin |
|  |  |  |
| Incorrect referral |  | Insubordinate referral to a psychiatrist |
|  |  |  |
| Incorrect/risky prescription of medicine |  | Prescribed an addictive drug with irreversible effects |
|  |  | Prescribing a drug with suicidality related side effects |
|  |  |  |
| Inexperienced practitioner |  | No adequate experience to treat the patient |
|  |  |  |
| Insufficient diagnostics about something other than STB |  | Did no try to examine alternative diagnoses |
|  |  |  |
| Insufficient record knowledge |  | Did not adequately delve into patient’s health status |
|  |  | Did not consult additional notes on the patient |
|  |  |  |

| Codebook for complaints – continued |  |  |
| --- | --- | --- |
| Code |  | Example from disciplinary case |
|  |  |  |
| Insufficient treatment of something other than STB |  | Carried out an inadequate treatment showing little expertise |
|  |  | Inadequate treatment of autism |
|  |  |  |
| Insufficient/inappropriate communication with the patient or system |  | Mentioned to the patient that it might be better to actually commit suicide |
|  |  |  |
| Misjudged suicide risk |  | Wrongly concluded that the patient was able to be dismissed from the hospital |
|  |  |  |
| Overtreatment |  | Relocated the patient, with no reason for imminent danger, to the locked ward. |
|  |  |  |
| Shared too much information with another health practitioner |  | Shared information with another health care practitioner without consent |
|  |  | Shared information with another health practitioner that was not directly involved with the patient |
|  |  |  |
| Too little cooperation with another health practitioner |  | Negligent in coordinating the treatment with the general practitioner |
|  |  |  |
| Too little cooperation with patient’s system |  | Did not take into account relevant information provided by the family of the patient |
|  |  |  |
| Insufficient care for surviving relatives |  | Did only consult with the patient’s relatives four days after the patient’s suicide |
|  |  | Did not provide surviving relatives’ care |
|  |  |  |
| Unacceptable behavior - personally involved with the patient |  | Transgressive behavior |
|  |  | Did not keep an appropriate social distance to the patient |
|  |  |  |
| Undertreatment |  | Did not provide the patient the necessary treatment |
|  |  | Did not oversee the patients’ needs during admission to the psychiatric hospital |
|  |  |  |
| Unpleasant physical examination |  | Heavy-handed physical examination resulting in mental distress |
|  |  |  |
| Used information from an unreliable source/person |  | Decision making on information received from health professionals unknown to the patient |
|  |  | Decision making based on an unoriginal document not related to the patient |
|  |  |  |

| Table A3. Codebook | | | | | | |
| --- | --- | --- | --- | --- | --- | --- |
| Disciplinary measure |  | Judgement |  | Profession |  | Work setting |
|  |  |  |  |  |  |  |
| Formal warning (appeal) |  | Founded (appeal) |  | Company doctor |  | Crisis team |
|  |  |  |  |  |  |  |
| Formal warning (first instance) |  | Founded (first instance) |  | General practitioner |  | General hospital |
|  |  |  |  |  |  |  |
| Repeal of measurement (appeal) |  | Unfounded (appeal) |  | Insurance physician |  | Immigration and Naturalization Service (INS) |
|  |  |  |  |  |  |  |
| Reprimand (first instance) |  | Unfounded (appeal) |  | Medical advisor |  | Inpatient |
|  |  |  |  |  |  |  |
| Revoked registration (appeal) |  |  |  | Nurse |  | Insurance company |
|  |  |  |  |  |  |  |
| Suspension (first instance) |  |  |  | Psychiatrist |  | Outpatient |
|  |  |  |  |  |  |  |
|  |  |  |  | Psychologist |  |  |
|  |  |  |  |  |  |  |
| Disciplinary tribunal |  | Lawyer |  | Suicidal thought or behavior* |  |  |
|  |  |  |  |  |  |  |
| Amsterdam (first instance) |  | Complainant with lawyer |  | Death by suicide |  |  |
|  |  |  |  |  |  |  |
| Central disciplinary tribunal (appeal) |  | Complainant without lawyer |  | Suicide attempt |  |  |
|  |  |  |  |  |  |  |
| The Hague (first instance) |  | Defendant with lawyer |  | Suicidal ideation |  |  |
|  |  |  |  |  |  |  |
| Eindhoven (first instance) |  | Defendant without lawyer |  |  |  |  |
|  |  |  |  |  |  |  |
| Groningen (first instance) |  |  |  |  |  |  |
|  |  |  |  |  |  |  |
| Zwolle (first instance) |  |  |  |  |  |  |
|  |  |  |  |  |  |  |

*During codification the following definitions were used. **Death by suicide:** An act resulting in death which is initiated and carried out by an individual to the end of the action, with the knowledge of a potentially fatal result, and in which intent may be ambiguous or unclear, may involve the risk of dying, or may not involve explicit intent to die. **Suicide attempt:** An act in which a person harms himself or herself, with the intention to die, and survives. **Suicidal ideation:** To think of suicide with or without suicidal intent, or hope for death by killing oneself, or state suicidal intention without engaging in behaviour. *Reference: De Leo D, Goodfellow B, Silverman M, et al. International study of definitions of English-language terms for suicidal behaviours: a survey exploring preferred terminology. BMJ Open 2021;11:e043409.*
